# Alcohol use polygenic risk score, social support, and alcohol use among European American and African American adults

**DOI:** 10.1017/S0954579423001141

**Published:** 2023-10-02

**Authors:** Jinni Su, Sally I-Chun Kuo, Fazil Aliev, Jill A. Rabinowitz, Belal Jamil, Grace Chan, Howard J. Edenberg, Meredith Francis, Victor Hesselbrock, Chella Kamarajan, Sivan Kinreich, John Kramer, Donbing Lai, Vivia McCutcheon, Jacquelyn Meyers, Ashwini Pandey, Gayathri Pandey, Martin H. Plawecki, Marc Schuckit, Jay Tischfield, Danielle M. Dick

**Affiliations:** 1 Department of Psychology, Arizona State University, Tempe, AZ, USA; 2 Department of Psychiatry, Rutgers University, New Brunswick, NJ, USA; 3 Bloomberg School of Public Health, Johns Hopkins University, Baltimore, MD, USA; 4 Department of Psychiatry, University of Connecticut, Farmington, CT, USA; 5 Department of Psychiatry, University of Iowa, Iowa City, IA, USA; 6 Department of Biochemistry and Molecular Biology, Indiana University, Indianapolis, IN, USA; 7 Department of Psychiatry, Washington University, St. Louis, MO, USA; 8 Department of Psychiatry, State University of New York Downstate Medical Center, Brooklyn, USA; 9 Department of Psychiatry, Indiana University, Bloomington, IN, USA; 10 Department of Psychiatry, University of California, San Diego, La Jolla, CA, USA; 11 Department of Genetics, Rutgers University, New Brunswick, NJ, USA; 12 Rutgers Addiction Research Center, Rutgers University, New Brunswick, NJ, USA

**Keywords:** polygenic scores, gene-environment interaction, social support, alcohol use, COGA

## Abstract

Alcohol use is influenced by genetic and environmental factors. We examined the interactive effects between genome-wide polygenic risk scores for alcohol use (alc-PRS) and social support in relation to alcohol use among European American (EA) and African American (AA) adults across sex and developmental stages (emerging adulthood, young adulthood, and middle adulthood). Data were drawn from 4,011 EA and 1,274 AA adults from the Collaborative Study on the Genetics of Alcoholism who were between ages 18–65 and had ever used alcohol. Participants completed the Semi-Structured Assessment for the Genetics of Alcoholism and provided saliva or blood samples for genotyping. Results indicated that social support from friends, but not family, moderated the association between alc-PRS and alcohol use among EAs and AAs (only in middle adulthood for AAs); alc-PRS was associated with higher levels of alcohol use when friend support was low, but not when friend support was high. Associations were similar across sex but differed across developmental stages. Findings support the important role of social support from friends in buffering genetic risk for alcohol use among EA and AA adults and highlight the need to consider developmental changes in the role of social support in relation to alcohol use.

In the U.S., alcohol use is common among adults and is associated with a number of negative social and health consequences, representing a significant public health concern (Grant et al., 2015). Genetics play an important role in alcohol use outcomes, accounting for approximately 50% of the variance in alcohol use disorders (Verhulst et al., 2015). Genetic influences vary as a function of environmental experiences, a phenomenon referred to as gene-environment interaction (G×E) (Plomin et al., 1977). That is, environmental factors can exacerbate or attenuate genetic influences on a given behavior. For example, environments that provide more social opportunity for or trigger alcohol use (e.g., affiliation with peers who use alcohol and substances, stressful life events) can exacerbate the effect of genetic risk for alcohol use, whereas environments that provide more social control against alcohol use (e.g., parental monitoring during adolescence) can attenuate genetic effects (Dick & Kendler, 2012). Understanding which environmental factors reduce alcohol use among those at greater genetic risk for alcohol use can inform prevention and intervention efforts aimed at mitigating risk for alcohol use and related negative sequelae.

Despite the fact that alcohol use is a health concern for all racial/ethnic groups in the United States, G×E research has primarily focused on populations of European ancestry. However, from a cultural genomics perspective (Causadias & Korous, 2018), genetically informed pathways of risk may vary across populations due to differences in genetic, environmental, and cultural factors and the complex interplay among them (Peterson et al., 2019). G×E effects in European American samples may not be generalizable to non-European populations (Su et al., 2018). Compared to European Americans (EA), African Americans (AA) on average consume lower levels of alcohol, but experience similar or higher levels of negative social and health consequences related to alcohol use (Mulia et al., 2009; Zapolski et al., 2014). AAs are more likely to have a lower socioeconomic status and experience stressful life events, such as racial discrimination (Williams et al., 2010), that are associated with increased alcohol use (Gilbert & Zemore, 2016). There are also important differences in genetic diversity, allele frequencies, and linkage disequilibrium patterns between EAs and AAs (Campbell & Tishkoff, 2008; Gabriel et al., 2002; Peterson et al., 2019; Rosenberg et al., 2010), potentially contributing to differences in the effects of specific genetic variants on phenotypes such as alcohol use. Taken together, G×E effects on alcohol use outcomes may vary across racial/ethnic groups; thus, it is important to study G×E processes in diverse populations to better understand pathways of risk and resilience related to alcohol use outcomes (Peterson et al., 2019).

Many G×E studies on alcohol use have used twin and family designs, and G×E studies with measured genotypes have traditionally focused on candidate genes or a single genetic polymorphism (Dick & Kendler, 2012). However, candidate G×E studies have been criticized for inconsistent results, which is likely due to the existence of publication bias, low statistical power, and a high false discovery rate (Dick et al., 2015; Duncan & Keller, 2011). Complex behaviors like alcohol use are polygenic, influenced by many genes of small effect sizes (Liu et al., 2019; Plomin et al., 2009). In recent years, there have been rapid advances in large scale genome-wide association studies (GWAS) to understand the complex genetic architecture underlying alcohol-related phenotypes (Gelernter et al., 2019; Kranzler et al., 2019; Liu et al., 2019; Walters et al., 2018; Zhou et al., 2020). To capitalize on the knowledge gained from GWAS, genome-wide polygenic scores have emerged as a powerful approach that aggregates information across individuals’ genome to characterize individuals’ genetic predispositions. The genome-wide polygenic score approach has been applied to understand associations between genetic predispositions and a variety of complex traits and behaviors (Bogdan et al., 2018), and can be leveraged to understand how genetic risk interacts with environmental factors to influence alcohol use problems (Dick et al., 2018). Prior research using the polygenic score approach has demonstrated significant polygenic effects on alcohol use outcomes among adolescents and adults in European American samples (Barr et al., 2020; Johnson et al., 2021; Schaefer et al., 2023). A few studies examined polygenic effects on alcohol use among African American adolescents and young adults but did not find significant associations (Elam et al., 2022; Su et al., 2022). One study examined polygenic effects on alcohol use from age 16 to 30, and found that polygenic risk for alcohol use was associated with alcohol use from age 22 to 27 among AAs and from age 24.5 to 29 among EAs, suggesting that polygenic effects on alcohol use may vary across age among EAs and AAs (Elam et al., 2021). A major limitation in the field is that GWAS and genetic research in general has been primarily focused on European Americans and other populations of European ancestry, but polygenic scores created based on a discovery GWAS in one racial/ethnic group have limited portability to and reduced predictive power in other racial/ethnic groups (Martin et al., 2019). This highlights the need for studies that develop alcohol use polygenic scores based on discovery GWAS in racial/ethnic samples aligned with those in the target sample, in order to improve accuracy and predictive power of polygenic scores in racial/ethnic minority groups.

One important environmental factor that may be particularly relevant in influencing alcohol use behaviors is social support. Social support is associated with decreases in a variety of risky behaviors, including decreasing levels of alcohol consumption and fewer alcohol-related problems among racially and ethnically diverse populations (Boateng-Poku et al., 2020; Cano et al., 2018). Social support may be protective by providing greater social control and/or buffering effects of stressors, thus reducing individuals’ likelihood of drinking to cope (Peirce et al., 1996). There is also evidence that social support can moderate genetic influences on substance use and that these associations may vary by sex and the source of social support (Barr et al., 2017; Jarnecke & South, 2014). For example, using a sample of young adults in the Finnish Twin Study, Barr and colleagues (2017) found that higher social support was associated with a decrease in genetic influence on alcohol use for men but an increase in genetic influences on alcohol use for women. In a sample of adult twins participating in the Midlife Development in the United States study, social support from romantic partners (but not from family or friends) moderated the genetic influence on alcohol problems, such that greater perceived support from romantic partners was associated with increased genetic influences (Jarnecke & South, 2014). Molecular genetic studies have also showed that social support buffered genetic risk in relation to alcohol use and related outcomes. For example, support from parents mitigated the genetic risk effect of the serotonin transporter gene (*5-HTTLPR*) on developmental trajectories of alcohol use from adolescence to young adulthood in a nationally representative sample (Su et al., 2019). Support from parents was found to attenuate the association between *5-HTTLPR* and depressive symptoms (Reinelt et al., 2015) and social anxiety disorders (Reinelt et al., 2014) among adults. A recent study using data from the Collaborative Study on the Genetics of Alcoholism (COGA) found that social support from friends buffered the effect of alcohol use polygenic scores on alcohol use among European American adults (Su et al., 2021); however, this study did not examine interactions between polygenic risk and social support among African Americans.

Alcohol use is a developmental phenomenon, where initiation typically occurs in adolescence and the risk for developing alcohol use disorder peaks in early adulthood (Grant et al., 2015). Twin studies suggest that genetic influences on alcohol use become increasingly important from adolescence to adulthood (Dick et al., 2007; Kendler et al., 2008). Molecular genetic studies have also shown that the effects of specific genes on alcohol use can vary across stages of development. For example, variation in the *GABRA2* genotype was associated with an increase in drunkenness that occurred at the transition between adolescence and adulthood (18–19 years), but not during adolescence (Dick et al., 2014). The importance of different environmental influences can also vary across development. For example, prior research suggests that peers and friends exert their most robust influence during adolescence and emerging adulthood (Harris, 1995; Hill et al., 2007). The nature and functioning of social support change across the life course (Ertel et al., 2009), with some evidence suggesting that support from friends may impact younger adults more and support from family may benefit older adults more (Walen & Lachman, 2000). Collectively, these findings suggest that genetic and environmental (e.g., social support) influences on alcohol use may vary across development. However, we note that in general there is a limited understanding of how genetic and G×E effects vary across developmental stages.

The overarching goal of the present study was to examine whether social support buffers genetic risk in relation to alcohol use among EA and AA adults and across men and women using a genome-wide polygenic score approach. We considered the effects of social support from both family and friends on alcohol use, allowing us to examine whether the effects differed by sources of social support. Building on prior research, we hypothesized that (1) higher alcohol use polygenic scores (alc-PRS) would be associated with higher levels of alcohol use in both EA and AA adults, and (2) higher social support would be associated with lower alcohol use in both EA and AA adults. Although prior twin studies yielded mixed findings regarding the moderating effect of social support on genetic influences (Barr et al., 2017; Jarnecke & South, 2014), molecular genetic studies have consistently demonstrated that social support may buffer genetic risk in relation to alcohol use and related outcomes (Reinelt et al., 2015; Su et al., 2019, 2021). Thus, we hypothesized that social support would moderate the association between alc-PRS and alcohol use such that this association would be weaker when social support is higher in EA and AA adults. In addition, we tested potential sex differences in the associations between alc-PRS, social support, and alcohol use, given evidence of sex differences in rates of alcohol use and levels of social support (Grant et al., 2015; Turner, 1994) and in view of recommendations for examining sex differences in G×E processes in relation to alcohol use outcomes and other traits (Martin et al., 2021; Salvatore et al., 2017). Finally, given prior evidence suggesting that genetic and environmental influences on alcohol use vary across development (Dick et al., 2007; Kendler et al., 2008), we capitalized on the rich dataset from the Collaborative Study on the Genetics of Alcoholism (COGA), which includes participants of a wide age range, to explore whether the associations between alc-PRS, social support, and alcohol use vary across developmental stages (i.e., emerging adulthoods [age 18–29] vs young adulthood [age 30–44] vs middle adulthood [age 45–65]). However, given that prior literature has not systematically examined developmental differences in effects of genetic factors, social support, and their interactions across adulthood, we did not have strong-enough evidence to derive specific hypotheses about how the associations between alc-PRS, social support, and alcohol use vary across developmental stages. We note that these developmental analyses are exploratory. Hypotheses of the current study were preregistered in the Open Science Framework (https://osf.io/hz7gb).

## Method

### Sample

Data for this study were drawn from the Collaborative Study on the Genetics of Alcoholism (COGA), a multi-site, large, multigenerational family study that aims to identify genetic influences on alcohol dependance and related psychiatric phenotypes (Begleiter et al., 1995; Edenberg, 2002). Probands were identified through alcohol treatment programs at seven U.S. sites and were invited to participate if they had a sufficiently large family (usually sibships of more than three with parents available) with two or more members in the COGA catchment areas. Comparison families were also recruited from the community. Data collection for COGA started in 1991 (Phase I) when adults in the target extended families were invited to complete the Semi-Structured Assessment for the Genetics of Alcoholism, a comprehensive interview that assesses demographic factors, alcohol use disorders, and a variety of psychiatric phenotypes (Bucholz et al., 1994). Participants were followed up about 5 years after they completed the first assessment (Phase II). The average time to follow up at Phase II was 5.7 years. Participants also completed questionnaires, including the Perceived Social Support from Family/Friends Scale at Phase II. COGA participants were asked to provide a DNA sample via blood or saliva. Institutional Review Boards at all sites approved this study, and written consents were obtained from participants.

For the purpose of the present study, we used data from COGA Phase II interviews, including measures of social support, to address our primary research questions as that is when social support was measured. We focused on COGA participants of European or African ancestry as determined by their genetic information who also self-identified as European American or Black/African American, respectively. We included participants who (1) were between ages 18–65 at the Phase II assessment, (2) had genomic data available for the calculation of alc-PRS, and (3) indicated that they had ever used alcohol. Our analytic sample included 4,011 (mean age = 38.43, SD = 11.96, 54.5% female) EAs and 1,274 (mean age = 35.97, SD = 11.11, 54.8% female) AAs. Results from independent sample t-tests indicated that individuals who were included in the analytic sample of the present study reported more drinks per week (*p* < .001) and were younger (*p* < .001) compared to those who completed the COGA Phase II assessment but were not included in the present study; however, they did not differ in terms of sex, educational attainment, and household income. About 79.1% of the COGA Phase II sample provided a DNA sample for genotyping. Those who provided a DNA sample did not differ significantly from those who did not in terms of alcohol use and social support (*p* > .05).

### Measures

#### Alcohol use

Alcohol use was measured as the number of drinks consumed in a typical week in the past 6 months (Bucholz et al., 1994). Participants reported the number of drinks of different kinds of alcoholic beverages (beer, wine, liquor, other) they consumed on a typical day of the week (Monday, Tuesday, … Sunday) over the past 6 months. Scores were summed across days and kinds of drinks to derive a composite score that indicated standard drinks per week. A total of 966 (24.1%) EA participants and 340 (26.7%) AA participants indicated that they consumed no alcohol (zero drinks) during the past 6 months and they were coded as zero. Extreme values (> 140 drinks per week; *n* = 24 (0.6%) for EAs and *n* = 12 (0.9%) for AAs) were winsorized (Keselman et al., 2008). Preliminary analysis indicated that the alcohol use variable was positively skewed (skewness = 4.19 and 3.42 for EAs and AAs, respectively), and thus was log transformed for subsequent regression analyses. The skewness for the log-transformed alcohol use variable was .65 and .69 for EAs and AAs, respectively.

#### Social support

Participants completed the Perceived Social Support from Family Scale and the Perceived Social Support from Friends Scale (Procidano & Heller, 1983). This is a 20-item self-report measure used to assess social support from family members or friends. Sample items include “My family/friends give me the moral support I need,” and “I rely on my family/friends for emotional support.” Participants responded to each statement on a 4-point scale ranging from 1 (generally false) to 4 (generally true). Scores were averaged across items; higher scores represent higher levels of support. Cronbach’s alpha was .95 and .93 for family support, and .91 and .86 for friend support, for EAs and AAs, respectively.

#### Genotyping and genome-wide polygenic scores

Participants’ DNA samples were genotyped using the Illumina Human1M array (Illumina, San Diego, CA), the Illumina Human OmniExpress 12V1 array, the Illumina 2.5M array, or the Smokescreen genotyping array (Biorealm LLC, Walnut, CA; Baurley et al., 2016). Data processing, quality control and imputation have been described elsewhere (Lai et al., 2019). Genotypes were imputed to 1000 Genomes using the cosmopolitan reference panel (Phase 3, Version 5, NCBI GRCh37) using SHAPPEIT2 and Minimac 3. Single nucleotide polymorphisms (SNPs) with a genotyping rate < 0.95, that violated Hardy-Weinberg equilibrium (*p* < 10^−6^) or had a minor allele frequency < 0.01, were excluded from analysis. More details on genotyping, quality control, and imputation for the COGA sample has been reported elsewhere (Wetherill et al., 2019).

The predictive power and accuracy of PRS depends largely on the statistical power of the discovery GWAS and the genetic ancestral similarities between the discovery and target samples (Duncan et al., 2019; Martin et al., 2019; Peterson et al., 2019). We calculated alc-PRS for EAs using estimates from a GWAS of alcohol use (drinks per week) in nearly 1 million individuals of European ancestry from the GWAS and Sequencing Consortium of Alcohol and Nicotine Use (Liu et al., 2019), one of the largest GWAS on alcohol-related phenotypes among EAs to date. We constructed alc-PRS for EAs using the PRS-CS method, a Bayesian regression and continuous shrinkage prior method shown to improved predictive power above traditional methods of PRS construction (Ge et al., 2019). An average of 2,069,117 SNPs was included in the construction of alc-PRS for EAs. For AAs, we used estimates from the GWAS of alcohol consumption (AUDIT-C) in the Million Veterans Project (MVP) sample (Kranzler et al., 2019), the largest published GWAS of alcohol phenotypes with a multiancestry sample including 209,020 EAs and 57,340 AAs. We calculated alc-PRS for our AA subsample using PRS-CSx, an extension of the PRS-CS method that integrates GWAS summary statistics from multiple populations and leveraging linkage disequilibrium (LD) diversity across discovery samples to improve polygenic prediction in non-EA samples (Ruan et al., 2022). Given GWAS summary statistics and ancestry-matched LD reference panels, PRS-CSx calculates one polygenic score for each discovery sample, and integrates them by learning an optimal linear combination to produce the final PRS. For the AA sample in the present study, Alc-PRS was calculated using PRS-CSx with GWAS summary statistics from the MVP EA and AA samples and 1000 Genomes ancestry-matched reference panels. An average of 865,352 SNPs was included in the construction of alc-PRS for AAs.

Consistent with prior research, we account for the first 10 genetic ancestry principal components (PC1-PC10) to adjust for potential population stratification. Specifically, we created residualized alc-PRS scores that account for PC1-PC10. The residualized alc-PRS scores were then standardized by creating Z-scores for subsequent analyses to aid in interpretation of results.

#### Covariates

Participants’ self-reported age, educational attainment, and household income, as well as sex recorded as observed by the interviewer, were included as covariates, given their demonstrated associations with alcohol use (Collins, 2016; Grant et al., 2015). Participants reported their highest level of education by responding to the question “What is the highest grade in school you completed?” Scores were converted to the number of years typically required to complete that level of education and ranged from 0 to 17 years. Participants also reported on their current household gross income based on a 9-point scale ranging from 0 (none) to 9 ($150,000 or more per year). Given the wide range of age in our sample, we created an age group variable to classify participants into three groups: emerging adulthood (ages 18–29, *n* = 1,068 EA and *n* = 390 AA), young adulthood (ages 30–44, *n* = 1,772 EA and *n* = 626 AA), and middle adulthood (ages 45–65, *n* = 1,234 EA and *n* = 275 AA). These age groups are consistent with studies in the epidemiology literature (e.g., Grant et al., 2015). This allowed us to examine similarities and differences in genetic and environmental influences across developmental periods.

### Analytic strategy

We first conducted preliminary analyses to examine descriptive statistics and correlations between study variables using SPSS 25.0. ANOVA analyses were also conducted to examine mean differences in social support and alcohol use across developmental stages. All analyses were stratified by ancestry. To test our hypotheses, we conducted a series of hierarchical linear regression analyses using Mplus version 8.3 separately for EAs and AAs. We included participants’ age, sex, educational attainment, and household income as covariates in all analyses. We first examined the main effects of alc-PRS on alcohol use (Model 1). Next, we added social support from family and friends as additional predictors to the regression model to examine main effects of family and friend support simultaneously (Model 2). To examine interaction effects between alc-PRS and family/friend support, we added product terms of alc-PRS and mean-centered family and friend support as additional predictors to the regression model (Model 3). We also examined potential gene-environment correlations (rGE) by testing correlations between alc-PRS and family/friend support. Significant rGE were accounted for in our models testing G×E effects by specifying alc-PRS and family/friend support to be correlated using the “WITH” command in Mplus. To check the robustness of significant G×E effects, we conducted additional analysis that included alc-PRS × covariate as well as social support × covariate interaction terms in the regression model to further account for potential confounding effects, following recommendations by Keller (2014).

We conducted multigroup analyses to examine whether the patterns of associations differed across sex and age groups by removing sex from the model and then comparing a model with the coefficients of interest constrained to equality with another model that had all coefficients freely estimated across females and females. Multigroup analyses across age groups were conducted by removing age from the model and then comparing a model with the coefficients of interest constrained to equality with another model that had all coefficients freely estimated across age groups (emerging adulthood vs young adulthood vs middle adulthood). A statistically significant Wald chi-square test of parameter equalities would indicate significant differences in regression coefficients across groups. Full information maximum likelihood estimation method was used to account for missing data and clustering within families was accounted using the CLUSTER command in Mplus. To account for the fact that we tested the associations independently in two ancestral groups, we used Bonferroni corrected *p* < .025 to evaluate statistical significance.

Finally, we conducted two sets of supplemental sensitivity analyses to further evaluate our findings. First, although the GSCAN GWAS sample is ideal for creating alc-PRS for our EA subsample given that it is the largest GWAS of alcohol phenotype (drinks per week) which also matched the alcohol phenotype of the present study, we recognize that the GSCAN sample is much larger than the MVP sample that we used to creating alc-PRS for AAs, which may be contributing to our finding of significant effects of alc-PRS in EAs but not AAs. Thus, we created alc-PRS for EAs using GWAS estimates from the MVP EA sample and ran analyses using this alc-PRS for EAs. Second, we evaluated developmental differences in G×E effects across emerging, young, and middle adulthood by creating three age bands that represent theoretically defined developmental periods (ages 18–29, 30–44, and 45–65). However, we recognize that these age bands are relatively large, and that there are vast differences within each age band concerning alcohol use. For example, alcohol use at age 18 is riskier and more problematic than at age 29. Thus, we conducted analyses using smaller age bands to further evaluate the developmental G×E effects. Given limitations by sample size, we created six smaller age bands. Specially, we split the “emerging adults” group into two groups: one group (ages 18–21) at or below the legal drinking age and another group (ages 22–29) above the legal drinking age; we further split the “young adulthood” and “middle adulthood” groups into two groups, cut at the middle of the age bands: (30–37 and 38–44 for young adults, 45–55 and 56–65 for middle adults). We note that creation of these smaller age bands is atheoretical and these analyses are exploratory.

## Results

### Preliminary analysis

Descriptive statistics and correlations between variables are presented in Table 1. Alc-PRS was positively correlated with alcohol use among EAs but not AAs. Both family and friend support were negatively correlated with alcohol use among EAs and AAs. Alc-PRS was negatively correlated with family and friend support among EAs. Results from ANOVA analyses indicated significant mean differences in social support and alcohol use across developmental stages (Supplemental Table 1).

### Predicting alcohol use from alc-PRS and social support

#### European Americans

Results from hierarchical multiple regression models predicting alcohol use are presented in Table 2. For EAs, there was a significant main effect of alc-PRS such that higher alc-PRS was associated with higher levels of alcohol use, above and beyond the effects of covariates (Model 1). Higher social support from friends was associated with lower alcohol use, but social support from family was not significantly associated with alcohol use (Model).

**Figure 1.**
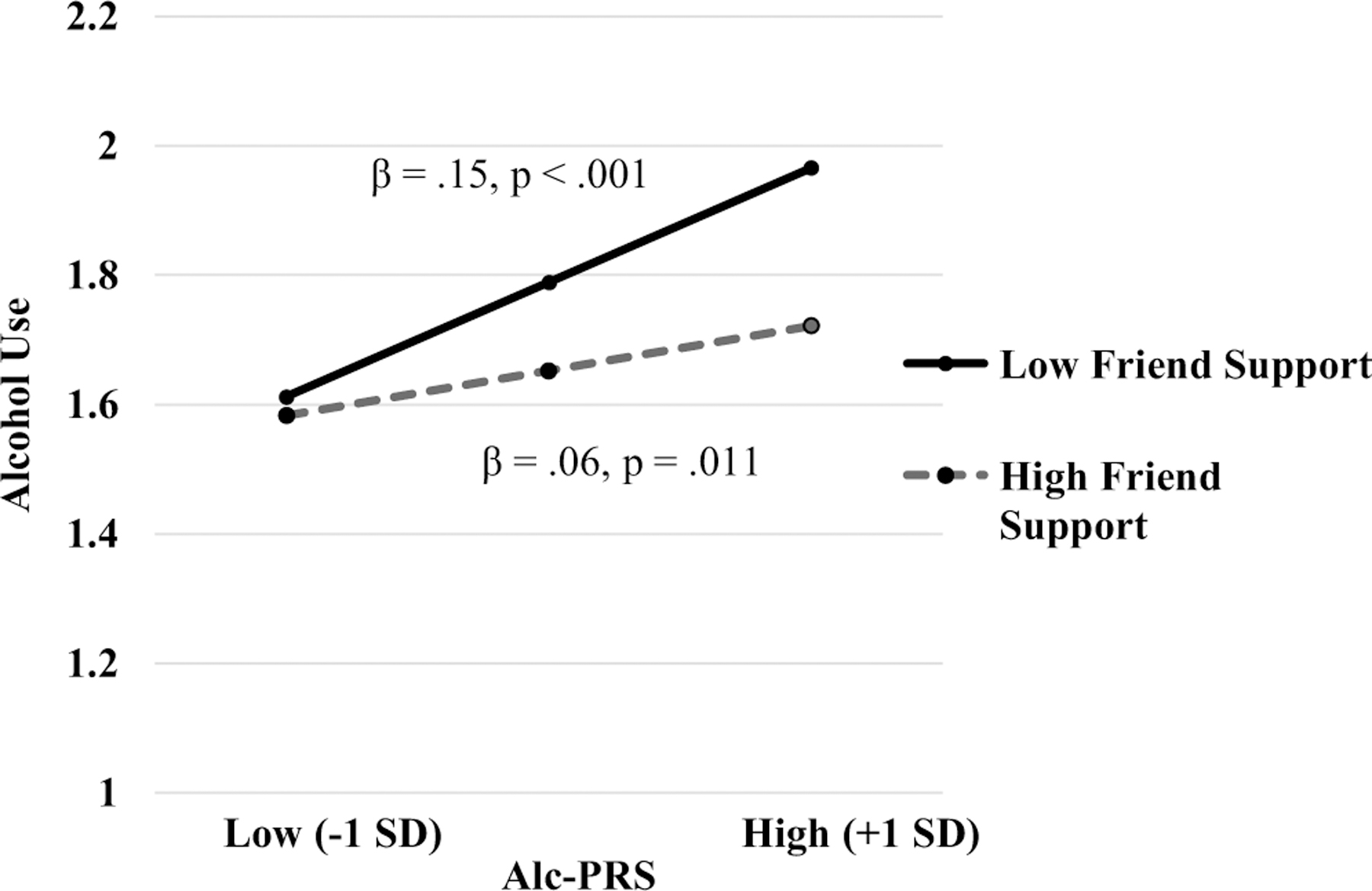
Alc-PRS by friend support interaction in relation to alcohol use among European Americans. Predicted values of log-transformed alcohol use (drinks per week) are plotted at prototypical values (+1/−1 *SD*) of alc-PRS and friend support.

There was an interaction between alc-PRS and friend support in predicting alcohol use (Model #). As illustrated in Figure 1, simple slope analysis indicated that alc-PRS was more strongly associated with alcohol use when friend support was low (−1 SD; *B* = .17, SE = .03, *β* = .14, *p* < .001) than when friend support was high (+1 SD; *B* = .07, SE = .03, *β* = .06, *p* = .011). The interaction effect between alc-PRS and friend support in relation to alcohol use remained statistically significant in follow up analysis where alc-PRS by covariate (age, sex, educational attainment, household income) and friend support by covariate interaction terms were included in the regression model (*p* = .013, see Supplemental Table 1), suggesting robustness of the interaction effect. There was no significant interaction between alc-PRS and family support in relation to alcohol use.

##### African Americans.

Consistent with findings for EAs, higher social support from friends was also associated with lower alcohol use, but social support from family was not significantly associated with alcohol use among AAs. However, there was no significant main effect of alc-PRS on alcohol use. In addition, we did not find any significant interactions between alc-PRS and social support from family or friends in relation to alcohol use in the AA subsample (see Table 3).

### Examining differences across developmental stages

#### European Americans

There were significant differences in the associations between social support and alcohol use across different developmental stages in adulthood for EAs (Table 4). Specifically, higher family support was associated with lower alcohol use (*B* = −.14, CI [−.23, −.06], SE = .05, *β* = −.09, *p* = .007) for emerging adults (age 18–29), higher alcohol use (*B* = .13, CI [.04, .22], SE = .05, *β* = .07, *p* = .013) for young adults (age 30–44), and was not significantly associated with alcohol use in middle adulthood (age 45–65; *B* = .03, CI [−.08, .13], SE = .06, *β* = .02, *p* = .671) (χ^2^ = 13.81, df = 2, *p* = .001). These associations significantly differed between emerging adults and young adults (χ^2^ = 13.61, df = 1, *p* < .001), but did not significantly differ between young adults and middle-aged adults (χ^2^ = 1.62, df = 1, *p* = .203) or between emerging adults and middle-aged adults (χ^2^ = 4.31, df = 1, *p* = .038). The association between friend support and alcohol use also varied across adulthood (χ^2^ = 17.94, df = 2, *p* < .001). Greater social support from friends was associated with higher alcohol use in emerging adulthood (*B* = .17, CI [ .05, .28], SE = .07, *β* = .08, *p* = .018), but lower alcohol use in young adulthood (*B* = −.22, CI [−.32, −.11], SE = .06, *β* = −.10, *p* < .001) and middle adulthood (B = −.18, [−.29, −.07], SE = .07, *β* = −.09, *p* = .008). These associations significantly differed between emerging adults and young adults (χ^2^ = 16.05, df = 1, *p* < .001) and between emerging adults and middle-aged adults (χ^2^ = 11.92, df = 1, *p* < .001), but did not differ between young adults and middle-aged adults (χ^2^ = .16, df = 1, *p* = .687). There were no significant differences in the effect of alc-PRS in relation to alcohol use across developmental stages. The interaction between alc-PRS and social support from friends was significant in young adulthood but not significant in emerging or middle adulthood; the interaction effect significantly differed between young adulthood and middle adulthood (χ^2^ = 6.85, df = 1, *p* = .009) but did not differ significantly between emerging and young adulthood (χ^2^ = 2.18, df = 1, *p* = .140) or between emerging and middle adulthood (χ^2^ = .57, df = 1, *p* = .452).

#### African Americans

As presented in Table 5, there were significant differences in the association between friend support and alcohol use across developmental stages for AAs. Specifically, friend support was significantly associated with lower alcohol use in emerging adulthood (*B* = −.28, CI [−.48, −.08], SE = .12, *β* = −.14, *p* = .023) and middle adulthood (*B* = −.59, CI [−.83, −.35], SE = .15, *β* = −.24, *p* < .001), but not in young adulthood (*B* = −.05, CI [−.22, .13], SE = .11, *β* = −.02, *p* = .667). These associations significantly differed between young adults and middle-aged adults (χ^2^ = 7.97, df = 1, *p* = .005), but there were no statistically significant difference in the associations between emerging adults and young adults (χ^2^ = 2.03, df = 1, *p* = .154) or between emerging adults and middle-aged adults (χ^2^ = 2.57, df = 1, *p* = .109).

**Figure 2.**
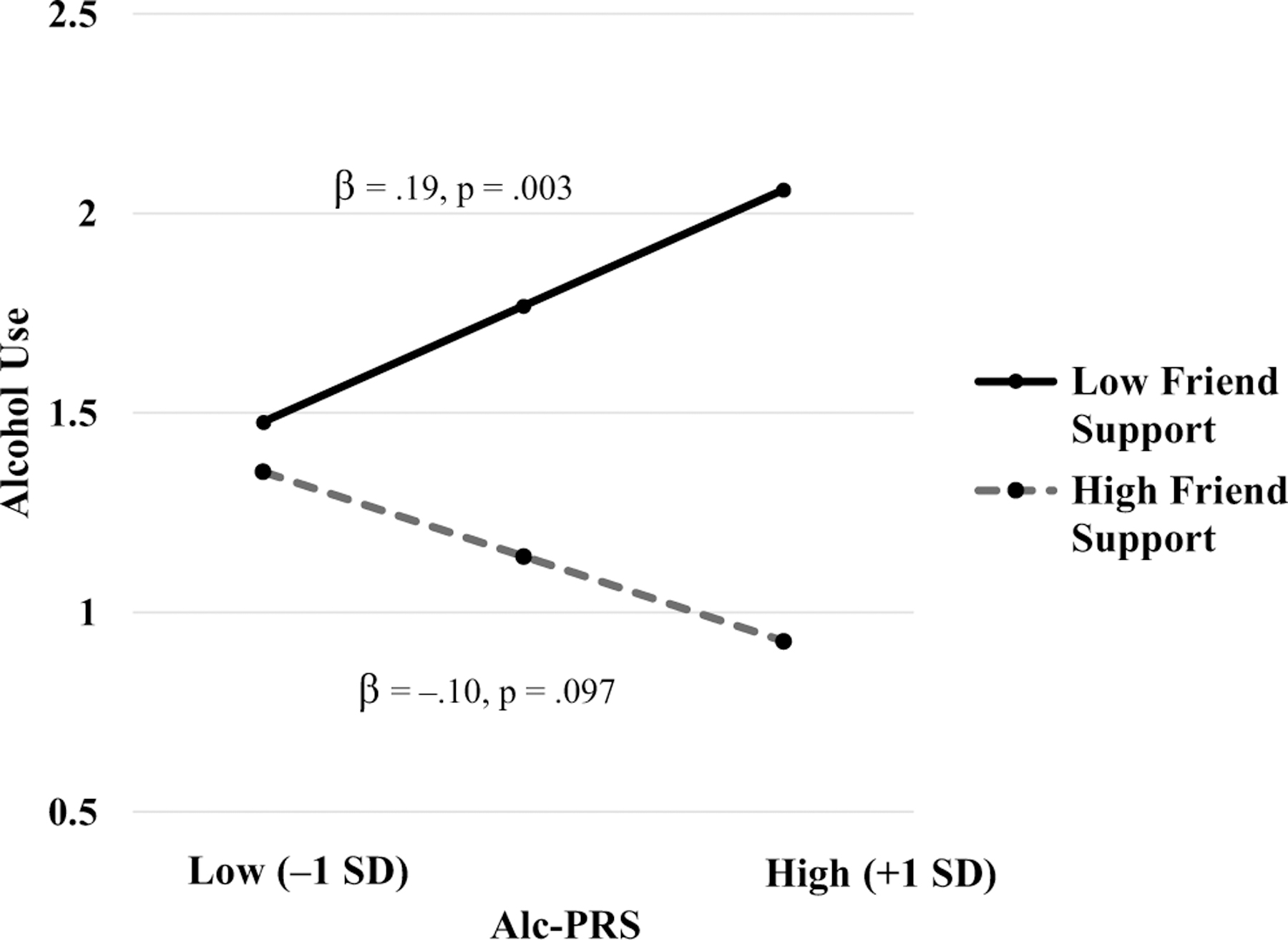
Alc-PRS by friend support interaction in relation to alcohol use among African Americans in middle adulthood. Predicted values of log-transformed alcohol use (drinks per week) are plotted at prototypical values (+1/−1 *SD*) of alc-PRS and friend support.

There were also significant differences in the interaction between alc-PRS and friend support in relation to alcohol use across developmental stages. This interaction between alc-PRS and friend support was significantly associated with alcohol use in middle adulthood (*B* = −.40, CI [−.56, −.24], SE = .10, *β* = −.18, *p* < .001), but not in emerging adulthood or young adulthood. These associations significantly differed between young adults and middle-aged adults (χ^2^ = 7.05, df = 1, *p* = .008) and between emerging adults and middle-aged adults (χ^2^ = 7.37, df = 1, *p* = .007), but did not significantly differ between emerging adults and young adults (χ^2^ = .29, df = 1, *p* = .593). As illustrated in Figure 2, simple slope analysis indicated that alc-PRS was significantly associated with alcohol use among middle-aged AA adults when friend support was low (−1 SD; *B* = .24, SE = .10, *β* = .16, *p* = .018), but not when friend support was high (+1 SD; *B* = −.18, SE = .09, *β* = −.13, *p* = .035). This interaction effect remained statistically significant after accounting for alc-PRS × covariate and friend support × covariate interactions (*p* = .007, see supplemental Table 2).

There were no significant differences in the associations between alc-PRS, family support, as well as the interaction between alc-PRS and family support, and alcohol use across developmental age groups.

### Examining sex differences

Results from multigroup analyses indicated that there was no significant difference in the associations between alc-PRS, social support, and their interactions, in relation to alcohol use between males and females for EAs (Walt test χ^2^ = 11.09, df = 5, *p* = .05; see Supplemental Table 3) or AAs (Walt test χ^2^ = 5.23, df = 5, *p* = .39; see Supplemental Table 4).

### Supplemental sensitivity analyses

Results from analyses with alc-PRS calculated based on GWAS summary statistics with the MVP EA sample were generally consistent with results presented above for the EA subsample, except that the interaction effect between alc-PRS and friend support was not significant. The main effect of alc-PRS on alcohol use was significant, although the effect size was relatively smaller in the sensitivity analysis, which may have hindered the detection of G´ E effects. Detailed results from this set of sensitivity analyses are presented in Supplemental Tables 6–8. Results from the sensitivity analyses with smaller age bands are presented in Supplemental Tables 9 and 10. Results are largely consistent with those conducted with larger age bands, except that the alc-PRS by friend support interaction significantly differed across developmental periods among EAs in the sensitivity analysis. The interaction is significant during ages 30–37 and the effect differed from those in other developmental periods. In addition, the main effect of friend support on alcohol use did not significantly differ across developmental periods among AAs in the sensitivity analysis, which could be due to smaller sample sizes with the smaller age bands and reduced statistical power.

## Discussion

The overarching goal of this study was to examine the interactive effects between genetic risk and social support in relation to alcohol use among EA and AA adults, using a genome-wide polygenic risk score approach. Utilizing data from a large diverse sample of adults from extended families enriched for alcohol use disorders, our findings indicated that perceived social support from friends buffers the effect of genetic risk for alcohol use on phenotypic alcohol use among both EA and AA (middle adulthood only for AAs) adults. We found no significant difference in the role of alc-PRS and social support in relation to alcohol use between males and females; however, important developmental differences in the role of social support in relation to alcohol use were observed among both EAs and AAs. This study extends the literature by taking a polygenic score approach to examine developmental G×E effects among both EA and AA adults, contributing novel insights regarding interactions between polygenic risk and social support in relation to alcohol use across ancestral groups, developmental stages, and sex.

Consistent with our hypothesis, higher alc-PRS was associated with greater alcohol use among EAs. Social support from friends (but not family) was associated with lower levels of alcohol use among EAs in the whole sample. In addition, we found evidence of an interaction effect between alc-PRS and perceived social support from friends in relation to alcohol use, such that high levels of friend support attenuated/buffered the association between alc-PRS and alcohol use. Our finding is consistent with prior research conducted in EAs indicating a buffering effect of social support on measured genetic risk (Reinelt et al., 2015; Su et al., 2019). Previous studies using twin-designs have also demonstrated interaction effects between genetic factors and social support; however, some studies found that genetic influences were stronger when social support was higher (Jarnecke & South, 2014). Barr et al. (2017) found that genetic influences on alcohol use were stronger when social support was higher for women, and the opposite was found for men. Differences in G×E findings across studies could be attributed to differences in genetic methodology, sample design, and measurement of constructs. By using a molecular genetic design, we characterized a specific dimension of genetic risk (i.e., alc-PRS), whereas twin studies quantify overall latent genetic influences. It is possible that high social support buffers genetic risk in relation to a particular developmental outcome, while also providing a context for the overall genetic predisposition to be expressed (rather than being suppressed). Future research is needed to replicate our findings and further understand the role of social support in moderating molecular genetic influences on alcohol use outcomes. Furthermore, we note that there was a small negative correlation between alc-PRS and family support and friend support. Although the present study focused on examining G×E effects, we note that these gene-environment correlation (rGE) processes may also underlie polygenic influences on alcohol use. Future research is warranted to examine rGE in addition to G×E processes to further understand mechanisms linking genetic risk and social support to alcohol use outcomes.

Similar to the findings for EAs, social support from friends (but not family) was associated with lower levels of alcohol use among AAs, suggesting that friend support is particularly important in adulthood. However, there was no significant main effect of alc-PRS on alcohol use among AAs. There was also no significant interaction effect between alc-PRS and social support from friends or family in relation to alcohol use among the whole AA subsample, which could be, at least in part, due to the low predictive ability of alc-PRS among AAs. This finding of limited predictive utility of polygenic scores among AAs is likely due to the underrepresentation and small sample size of AAs in the GWAS studies that the alc-PRS was derived from (Martin et al., 2019; Su et al., 2018). These findings highlight the continued need for increased representation of AAs in genetic research to better characterize genetic risk among this population. Additionally, we note that alc-PRS for AAs were generated for the AUDIT-C while alc-PRS for EAS were generated for drinks per week, which matched more closely to the outcome of the present study. Prior research indicates that PRS prediction is the strongest with matched phenotypes between the discovery GWAS and the target sample (Docherty et al., 2018). Thus, it is possible that the reduced predictability of alc-PRS among AAs in our sample could be due to the mismatch in phenotypes between the discovery GWAS and the current study.

Notably, prior G×E research on alcohol use outcomes primarily focused on adolescents and young adults, and limited research has examined G×E influences across a broader age range of adulthood (Su et al., 2021). This study examined potential developmental differences in the role of alc-PRS and social support in alcohol use across the adulthood years. For EAs, the main effect of alc-PRS on alcohol use appears to be fairly consistent across adulthood. This appears to be inconsistent with prior evidence that heritability of alcohol use increases from adolescence to adulthood (Dick et al., 2007; Kendler et al., 2008). The inconsistent findings may be due to different methodology (twin studies vs. polygenic scores). The limited predictability of alc-PRS in the present study may also have hindered the detection of developmental differences. Alternatively, this may be due to the fact that our sample was comprised of high-risk adult alcohol users, the majority of whom may have established their drinking patterns and thus genetic influences on their alcohol use may be more stable. Future research is needed to further examine developmental changes in polygenic effects on alcohol use. However, the role of social support on alcohol use varied across adulthood. Specifically, friend support was associated with more alcohol use in emerging adulthood, but was protective and associated with lower alcohol use in young and middle adulthood. This may be because alcohol use during emerging adulthood typically occurs in peer social settings (LaBrie et al., 2007). Thus, it is possible that support from friends during emerging adulthood may not provide increased social control in limiting alcohol use. Family support was most protective against alcohol use in emerging adulthood, but was associated with more alcohol use in young adulthood. This finding is consistent with prior research showing the continued protective role of parental support for emerging adults (Kouros et al., 2017; Serido et al., 2014). Young adults may be more likely to drink with family members (e.g., spouse) and thus a strong family support may be associated with more drinking in the family context during young adulthood (Homish & Leonard, 2007). Our preliminary analyses indicated significant mean-level differences in social support across developmental stages. However, there were no clear pattern of connection between differences in levels of social support and differences in the association between social support and alcohol use across developmental stages. Thus, it is unlikely that the observed differences in effects of social support across developmental stages are due to differences in endorsement of social support.

For AAs, family support was not associated with alcohol use across adulthood, whereas friend support was protective against alcohol use in emerging and middle adulthood, above and beyond the effect of age, sex, education, and family income. It is possible that for AAs family support plays a stronger role in alcohol use earlier in development (e.g., adolescence) than in adulthood. We note that family support was negatively correlated with alcohol use among AAs in bivariate correlation analyses. These findings are partially consistent with prior research in AA middle-aged adults, which found that both friend and family emotional support was negatively associated with alcohol use (Boateng-Poku et al., 2020). Our findings also indicated differences in the interaction between alc-PRS and friend support in relation to alcohol use across developmental stages. That is, friend support attenuated genetic risk for alcohol use during middle adulthood but not during emerging or young adulthood. These findings highlight the particularly important role of social support from friends during middle adulthood for AAs. To our knowledge, this is the first study to show developmental differences in the role of social support in moderating genetic influences among AAs. This interaction between alc-PRS and friend support in relation to alcohol use among AA middle adults remained statistically significant (*p* = .007) in the robustness analysis where alc-PRS × covariate and friend support × covariate interactions were included to further account for potential confounding effects, suggesting the robustness of the effect. However, we acknowledge that the sample size for the AA middle adults subsample is quite small (*n* = 273). With limited statistical power, it is possible that the detected interaction effect between PRS and friend support was a false positive finding. Thus, we consider our findings preliminary and future research is needed to replicate our findings.

Collectively, these results highlight the importance of examining developmental changes in the role of social support from family and friends in relation to alcohol use. Prior research suggests that the influence of social support on alcohol use outcomes may depend on the drinking behaviors of the social partners. Friends, romantic partners, and spouses who are more congruent in their drinking patterns tend to report higher levels of relationship satisfaction (Homish & Leonard, 2007; Marshal, 2003; Osgood et al., 2013). Those who perceive higher social support and have friends, romantic partners, or spouses who engage in high levels of drinking may be at higher risk for alcohol use. Future research is warranted to examine the role of social partners’ drinking behavior in moderating the association between social support and alcohol use across developmental stages.

Consistent with previous evidence of different levels of alcohol use and social support between males and females (Grant et al., 2015; Turner, 1994), we found that males reported lower levels of social support from family and friends and higher levels of alcohol use compared to females. However, despite these mean-level differences, we did not find any significant sex differences in the associations between alc-PRS, social support, and alcohol use among both EA and AA adults. This is consistent with prior findings suggesting that the effects of alc-PRS, social support, and their interactions on alcohol use are similar for males and females (Su et al., 2021). Nevertheless, future research is needed to further examine sex differences in G×E processes in genetically informed alcohol research (Salvatore et al., 2017). Additionally, although the present study is not powered to do so, future research is needed to examine G×E by sex by development effects on alcohol use and related outcomes, as sex differences in G×E effects may vary across development and vice versa.

There are several strengths of this study, including a relatively large sample size and use of the genome-wide polygenic score approach to characterize genetic risk. In addition, we included a sample of both EAs and AAs and our findings contribute to the limited G×E literature among non-European populations (Dick et al., 2017). Furthermore, our sample of a wide age range allowed for the examination of differences in the role of alc-PRS and social support in relation to alcohol use across emerging, young, and middle adulthood. Despite these strengths, our findings need to be interpreted in light of several limitations. First, our cross-sectional analyses preclude us from making causal inferences regarding the relation between social support and alcohol use. Second, our examination of differences across developmental periods based on these cross-sectional analyses may also be subject to bias due to cohort effects. Future research should employ longitudinal designs to examine within-person changes in the role of genetic risk and social support in relation to alcohol use across development. Third, despite using a relatively large sample size compared to many other prior G×E studies, the sample size for the AA subsample may still be underpower to detect 3-way interactions for testing sex differences or developmental differences in G×E effects. Future studies are needed to replicate the present findings with larger AA samples. Furthermore, COGA is a high-risk sample of participants from extended families enriched for alcohol use disorders and findings from this study may not be generalizable to other samples with different recruitment strategies (Savage et al., 2018). Thus, our sample is not representative of the general population, which may increase the potential for collider bias in estimating the effect of alc-PRS (Akimova et al., 2021). Future research is needed to replicate our findings in community and population-based samples and to apply advanced methodologies such as principal component analysis of measured cofounders as a method to reduce collider bias (Thomas et al., 2022). Finally, we note that the effect sizes for the significant genetic main and G × E effects are quite small. While these small effect sizes appear to run counter to the heritability estimates from quantitative genetic studies, they are consistent with findings from other studies using a polygenic score approach. These likely reflect methodological differences between the polygenic score approach and quantitative genetic approaches (which estimate the overall latent genetic influences on a phenotype). These findings also indicate limitations of the current polygenic score approach in predicting alcohol use outcomes, and highlight the need for improving predictability of polygenic scores with methodology advancements (e.g., larger discovery GWAS particularly among AAs, functional polygenic scores).

In conclusion, our findings show that perceived social support from friends play an important role in buffering the effects of genetic risk for alcohol use and suggest that the role of social support from both family and friends in relation to alcohol use vary across age. These results suggest that strengthening social support may be one means to reduce alcohol use among EA and AA adults. Given prior evidence that psychosocial interventions may mitigate genetic risk for alcohol use outcomes (Kuo et al., 2019; Neale et al., 2021), our findings also suggest that intervention programs that target at strengthening social support from friends may have the potential to reduce alcohol use among those high in genetic risk.

## Supplementary Material

1

## Figures and Tables

**Table 1.** Bivariate correlations among key study variables for European Americans and African Americans

European Americans								
	Age	Sex	Education	Income	Alc-PRS	Family Supp	Friend Supp	Alcohol use
Age	–							
Sex	.02	–						
Education	.07*	−.03	–					
Income	.15**	−.02	.30**	–				
Alc-PRS	.02	.02	−.02	−.01	–			
Family supp	.03	−.12**	.17**	.17**	−.05**	–		
Friend supp	−.14**	−.27**	.11**	.07**	−.03*	.41**	–	
Alcohol use	−.08**	.23**	−.17**	−.15**	.10**	−.13**	−.16**	–
N	4011	4011	4011	4011	4011	3650	3650	4011
Mean	38.43	.45^a^	13.36	4.55	0/0^b^	3.02	3.03	9.02
SD	11.96	–	2.27	2.75	1.00^b^	.67	.59	18.45
African Americans								
	Age	Sex	Education	Income	Alc-PRS	Family Supp	Friend Supp	Alcohol use
Age	–							
Sex	.02	–						
Education	.10**	−.11*	–					
Income	.15**	−.03	.31**	–				
Alc-PRS	−.02	.01	−.02	.01	–			
Family supp	.09**	−.03	.07*	.14**	.03	–		
Friend supp	−.01	−.14**	.15**	.12**	.02	.38**	–	
Alcohol use	.06*	.22**	−.16**	−.13**	−.01	−.09**	−.17**	–
N	1274	1274	1274	1274	1274	1159	1155	1274
Mean	35.97	.45^a^	12.10	2.69	0/0^b^	2.95	2.89	11.20
SD	11.11	–	1.89	3.07	1.00^b^	.67	.57	22.72

Alc-PRS = alcohol use genome-wide polygenic score; Supp = support; Sex is coded as 1 = male; 0 = female.

a proportion of males.

b alc-PRS are standardized. Descriptive statistics for raw scores of alcohol use are presented.

* *p* < .05.

** *p* < .01.

Means and standard deviations for variables are presented for European Americans/African Americans.

**Table 2.** Predicting alcohol use from alcohol use polygenic scores and social support among European American adults

	Model 1				Model 2				Model 3			
	B	SE	β	p	B	SE	β	p	B	SE	β	p
Age	−.02	.00	−.21	< .001	−.02	.00	−.21	< .001	−.02	.00	−.22	< .001
Sex	.54	.04	.22	< .001	.51	.04	.21	< .001	.51	.04	.21	< .001
Educational attainment	−.04	.01	−.07	< .001	−.04	.01	−.07	< .001	−.03	.01	−.06	< .001
Household income	−.02	.01	−.03	.056	−.01	.01	−.03	.067	−.01	.01	−.03	.061
Alc-PRS	**.12**	**.02**	**.10**	**< .001**	**.12**	**.02**	**.10**	**< .001**	.12	.02	.10	< .001
Family support					.03	.03	.02	.350	.03	.03	.02	.327
Friend support					**−.12**	**.04**	**−.06**	**.002**	**−.12**	**.04**	**−.06**	**.002**
Alc-PRS × Family support									−.04	.03	−.02	.313
Alc-PRS × Friend support									**−.09**	**.04**	**−.05**	**.018**

N = 4,011; Alc-PRS = alcohol consumption genome-wide polygenic score.

Statistically significant effects of Alc-PRS and social support are bolded.

**Table 3.** Predicting alcohol use from alcohol use polygenic scores and social support among African American adults

	Model 1				Model 2				Model 3			
	B	SE	β	p	B	SE	β	p	B	SE	β	p
Age	.00	.00	.00	.965	−.00	.00	−.00	.910	.00	.00	.00	.881
Sex	.69	.07	.26	< .001	.66	.07	.25	< .001	.66	.07	.25	< .001
Educational attainment	−.07	.02	−.10	.001	−.06	.02	−.09	.004	−.06	.02	−.09	.004
Household income	−.03	.01	−.07	.024	−.02	.01	−.06	.054	−.02	.01	−.05	.067
Alc-PRS	.00	.04	.00	.959	.01	.04	.00	.901	.01	.04	.01	.867
Family support					−.03	.07	−.02	.640	−.04	.07	−.02	.614
Friend support					**−.25**	**.07**	**−.11**	**< .001**	**−.25**	**.07**	**−.11**	**< .001**
Alc-PRS × Family support									.10	.07	.05	.117
Alc-PRS × Friend support									−.11	.07	−.05	.108

N = 1,274; Alc-PRS = alcohol consumption genome-wide polygenic score.

Statistically significant effects of social support are bolded.

**Table 4.** Predicting alcohol use from alcohol use polygenic scores and social support: testing developmental differences among European Americans adults

	Emerging adulthood (*n* = 1056)				Young adulthood (*n* = 1733)				Middle adulthood (*n* = 1222)				Wald tests	
	B	SE	β	p	B	SE	β	p	B	SE	β	p	χ^2^ (df = 2)	p
**Step 1**														
Sex	.69	.06	.32	< .001	.43	.06	.17	< .001	.52	.07	.22	< .001	–	–
Educational attainment	−.03	.02	−.07	.044	−.08	.02	−.13	< .001	.01	.02	.02	.585	–	–
Household income	.00	.01	.00	.974	−.04	.02	−.07	.017	−.02	.01	−.04	.212	–	–
Alc-PRS	.11	.04	.10	.003	.13	.03	.10	< .001	.12	.03	.11	< .001	.06	.972
**Step 2**														
Family support	**−.14**	**.05**	**−.09**	**.007**	**.13**	**.05**	**.07**	**.013**	**.03**	**.06**	**.02**	**.671**	**13.81**	**< .001**
Friend support	**.17**	**.07**	**.08**	**.018**	**−.22**	**.06**	**−.10**	**< .001**	**−.18**	**.07**	**−.09**	**.008**	**17.94**	**< .001**
**Step 3**														
Alc-PRS × Family support	−.06	.06	−.03	.354	.02	.06	.01	.748	−.10	.06	−.06	.075	2.21	.332
Alc-PRS × Friend support	−.04	.07	−.02	.558	−.18	.06	−.09	.002	.03	.06	.02	.623	6.98	.031

Alc-PRS = alcohol consumption genome-wide polygenic score.

Emerging adulthood ages 18–29, young adulthood ages 30–44, and middle adulthood ages 45–65. Coefficients that are statistically significantly different across age groups are bolded.

**Table 5.** Predicting alcohol use from alcohol use polygenic scores and social support: testing developmental differences among African American adults

	Emerging adulthood (*n* = 387)				Young adulthood (*n* = 614)				Middle adulthood (*n* = 273)				Wald tests	
	B	SE	β	p	B	SE	β	p	B	SE	β	p	χ^2^ (df = 2)	p
**Step 1**														
Sex	.63	.10	.28	< .001	.66	.12	.23	< .001	.89	.18	.31	< .001	–	–
Educational attainment	−.08	.03	−.13	.015	−.06	.04	−.08	.087	−.02	.04	−.03	.711	–	–
Household income	.02	.01	.07	.075	-.07	.02	-.14	.002	-.13	.05	-.20	.003	–	–
Alc-PRS	−.03	.05	−.03	.590	.03	.06	.02	.621	.02	.08	.01	.806	.65	.722
**Step 2**														
Family support	.01	.10	.01	.885	−.15	.10	−.07	.133	.18	.17	.08	.28	3.47	.176
Friend support	**−.28**	**.12**	**−.14**	**.023**	**−.05**	**.11**	**−.02**	**.667**	**−.59**	**.15**	**−.24**	**< .001**	**8.07**	**.018**
**Step 3**														
Alc-PRS × Family support	.08	.09	.05	.402	.08	.11	.04	.473	.20	.14	.09	.147	.78	.679
Alc-PRS × Friend support	**−.04**	**.10**	**−.02**	**.702**	**.04**	**.12**	**.02**	**.717**	**−.40**	**.10**	**−.18**	**< .001**	**9.62**	**.008**

Alc-PRS = alcohol consumption genome-wide polygenic score.

Emerging adulthood ages 18–29, young adulthood ages 30–44, and middle adulthood ages 45–65. Coefficients that are statistically significantly different across age groups are bolded.
