# Supplementary material for: Alcohol use polygenic risk score, social support, and alcohol use among European American and African American adults
Source: Dev Psychopathol. Author manuscript; Available in PMC 2025 Apr 2. (PMC10985050; doi:10.1017/S0954579423001141)
Supplement: 1 [file NIHMS1925896-supplement-1.docx]

Supplemental Table 1

Results from ANOVA Comparing Means of Social Support and Alcohol Use Across Developmental Stages

|  | Emerging Adulthood | Young Adulthood | Middle Adulthood | F-test (p) |
| --- | --- | --- | --- | --- |
| **European Americans** |  |  |  |  |
| Family Support | 3.05 (.67) ^a^ | 2.98 (.68) ^b^ | 3.06 (.64) ^a^ | 6.15 (.002) |
| Friend Support | 3.17 (.55) ^a^ | 3.01 (.60) ^b^ | 2.95 (.58) ^c^ | 41.37 (< .001) |
| Drinks per Week | 9.93 (16.53) ^a^ | 10.45 (23.59) ^a^ | 6.97 (19.11) ^b^ | 11.02 (< .001) |
| **African Americans** |  |  |  |  |
| Family Support | 2.89 (.68) ^a^ | 2.93 (.67) ^a^ | 3.07 (.64) ^b^ | 5.26 (.005) |
| Friend Support | 2.93 (.56) ^a^ | 2.84 (.57) ^a^ | 2.93 (.59) ^a^ | 3.25 (.039) |
| Drinks per Week | 8.31 (18.52) ^a^ | 13.89 (32.67) ^b^ | 11.85 (23.67) ^ab^ | 4.99 (.007) |

Supplemental Table 2

Testing Interaction Effects Between Alc-PRS and Friend Support in Relation to Alcohol Use Among European Americans: Accounting for G × covariate and E × covariate in Testing G×E Effects

|  | B | SE | β | p |
| --- | --- | --- | --- | --- |
| Age | -.02 | .00 | -.21 | < .001 |
| Sex | .51 | .04 | .21 | < .001 |
| Education | -.03 | .01 | -.06 | < .001 |
| Income | -.02 | .01 | -.03 | .049 |
| Alc-PRS | .16 | .13 | .14 | .210 |
| Family Support | .04 | .03 | .02 | .253 |
| Friend Support | .16 | .24 | .08 | .521 |
| Alc-PRS × Family Support | -.02 | .04 | -.01 | .480 |
| **Alc-PRS × Friend Support** | **-.10** | **.04** | **-.05** | **.013** |
| Alc-PRS × Age | .00 | .00 | -.00 | .939 |
| Alc-PRS × Sex | -.05 | .04 | -.03 | .228 |
| Alc-PRS × Education | .00 | .01 | .05 | .651 |
| Alc-PRS × Income | -.02 | .01 | -.07 | .032 |
| Friend Support × Age | -.01 | .00 | -.13 | .033 |
| Friend Support × Sex | -.08 | .07 | -.03 | .302 |
| Friend Support × Education | -.01 | .02 | -.07 | .521 |
| Friend Support × Income | .03 | .01 | .08 | .017 |

Note. N = 4,011. Alc-PRS = alcohol consumption genome-wide polygenic score. Statistically significant interaction effect between alc-PRS and friend support is bolded.

Supplemental Table 3

Testing Interaction Effects Between Alc-PRS and Friend Support in Relation to Alcohol Use Among African Americans in Middle Adulthood: Accounting for G × covariate and E × covariate in Testing G×E Effects

|  | B | SE | β | p |
| --- | --- | --- | --- | --- |
| Age | -.03 | .02 | -.13 | .029 |
| Sex | .85 | .18 | .30 | < .001 |
| Education | .00 | .04 | .01 | .924 |
| Income | -.15 | .05 | -.22 | .001 |
| Alc-PRS | -.47 | .78 | -.33 | .545 |
| Family Support | .18 | .16 | .08 | .263 |
| Friend Support | -1.12 | 1.28 | -.46 | .381 |
| Alc-PRS × Family Support | .18 | .15 | .08 | .224 |
| **Alc-PRS × Friend Support** | **-.34** | **.13** | **-.15** | **.007** |
| Alc-PRS × Age | .01 | .01 | .26 | .495 |
| Alc-PRS × Sex | .02 | .17 | .01 | .902 |
| Alc-PRS × Education | .01 | .03 | .06 | .846 |
| Alc-PRS × Income | .01 | .04 | .02 | .886 |
| Friend Support × Age | .02 | .02 | .44 | .339 |
| Friend Support × Sex | -.13 | .31 | -.03 | .685 |
| Friend Support × Education | -.08 | .07 | -.40 | .239 |
| Friend Support × Income | .17 | .09 | .27 | .042 |

Note. N = 273; Alc-PRS = alcohol consumption genome-wide polygenic score. Statistically significant interaction effect between alc-PRS and friend support is bolded.

Supplemental Table 4

Predicting Alcohol Use from Alcohol Use Genome-wide Polygenic Scores and Social Support: Testing Sex Differences among European American Adults

|  | Males (N = 1824) | | | | Females (N = 2187) | | | | Wald Test | |
| --- | --- | --- | --- | --- | --- | --- | --- | --- | --- | --- |
|  | B | SE | β | p | B | SE | β | p | χ^2^ (df = 1) | p |
| **Step 1** |  |  |  |  |  |  |  |  |  |  |
| Age | -.02 | .00 | -.21 | < .001 | -.02 | .00 | -.22 | < .001 | -- | -- |
| Educational Attainment | -.05 | .02 | -.08 | .003 | -.03 | .01 | -.05 | .028 | -- | -- |
| Household Income | -.03 | .01 | -.06 | .027 | -.00 | .01 | -.01 | .609 | -- | -- |
| Alc-PRS | .12 | .03 | .09 | < .001 | .13 | .02 | .13 | < .001 | .05 | .822 |
| **Step 2** |  |  |  |  |  |  |  |  |  |  |
| Family Support | .09 | .06 | .04 | .133 | -.00 | .04 | -.00 | .989 | 1.71 | .191 |
| Friend Support | -.20 | .06 | -.08 | .002 | -.06 | .05 | -.04 | .181 | 3.03 | .082 |
| **Step 3** |  |  |  |  |  |  |  |  |  |  |
| Alc-PRS x Family Support | -.07 | .06 | -.04 | .197 | .00 | .04 | .00 | .983 | 1.12 | .290 |
| Alc-PRS x Friend Support | -.16 | .07 | -.07 | .014 | -.05 | .05 | -.03 | .332 | 1.88 | .170 |

Note. Alc-PRS = alcohol consumption genome-wide polygenic score.

Supplemental Table 5

Predicting Alcohol Use from Alcohol Use Genome-wide Polygenic Scores and Social Support: Testing Sex Differences among African American Adults

|  | Males (N = 576) | | | | Females (N = 698) | | | | Wald Test | |
| --- | --- | --- | --- | --- | --- | --- | --- | --- | --- | --- |
|  | B | SE | β | p | B | SE | β | p | χ^2^ (df = 1) | p |
| **Step 1** |  |  |  |  |  |  |  |  |  |  |
| Age | .01 | .01 | .04 | .330 | -.01 | .00 | -.04 | .234 | -- | -- |
| Educational Attainment | -.08 | .04 | -.10 | .018 | -.07 | .03 | -.11 | .014 | -- | -- |
| Household Income | -.05 | .02 | -.10 | .013 | -.01 | .02 | -.03 | .469 | -- | -- |
| Alc-PRS | .03 | .06 | .02 | .636 | -.02 | .05 | -.02 | .687 | .41 | .524 |
| **Step 2** |  |  |  |  |  |  |  |  |  |  |
| Family Support | -.08 | .12 | -.03 | .540 | -.01 | .08 | -.01 | .890 | .25 | .621 |
| Friend Support | -.41 | .13 | -.15 | .001 | -.15 | .08 | -.08 | .073 | 3.02 | .083 |
| **Step 3** |  |  |  |  |  |  |  |  |  |  |
| Alc-PRS x Family Support | .14 | .11 | .06 | .191 | .08 | .07 | .05 | .296 | .29 | .592 |
| Alc-PRS x Friend Support | -.10 | .12 | -.04 | .412 | -.09 | .08 | -.05 | .266 | .00 | .951 |

Note. Alc-PRS = alcohol consumption genome-wide polygenic score.

Supplemental Table 6

Predicting Alcohol Use from Alcohol Use Polygenic Scores (MVP alc-PRS) and Social Support Among European American Adults

|  | Model 1 | | | | Model 2 | | | | Model 3 | | | |
| --- | --- | --- | --- | --- | --- | --- | --- | --- | --- | --- | --- | --- |
|  | B | SE | β | p | B | SE | β | p | B | SE | β | p |
| Age | -.02 | .00 | -.20 | <.001 | -.02 | .00 | -.21 | <.001 | -.02 | .00 | -.22 | <.001 |
| Sex | .54 | .04 | .22 | <.001 | .51 | .04 | .21 | <.001 | .51 | .04 | .21 | <.001 |
| Educational Attainment | -.04 | .01 | -.08 | <.001 | -.04 | .01 | -.07 | <.001 | -.04 | .01 | -.06 | <.001 |
| Household Income | -.02 | .01 | -.04 | .038 | -.02 | .01 | -.04 | .049 | -.02 | .01 | -.03 | .045 |
| Alc-PRS | **.09** | **.02** | **.08** | **<.001** | **.10** | **.02** | **.08** | **<.001** | **.09** | **.02** | **.10** | **<.001** |
| Family Support |  |  |  |  | .03 | .03 | .02 | .425 | .03 | .03 | .02 | .429 |
| Friend Support |  |  |  |  | **-.13** | **.04** | **-.06** | **.001** | **-.13** | **.04** | **-.06** | **.001** |
| Alc-PRS x Family Support |  |  |  |  |  |  |  |  | -.03 | .03 | -.02 | .439 |
| Alc-PRS x Friend Support |  |  |  |  |  |  |  |  | -.00 | .04 | -.05 | .984 |

Note. Alc-PRS = alcohol consumption genome-wide polygenic score calculated using GWAS estimates from the MVP EA sample. Statistically significant (p < .025) effects of Alc-PRS and social support are bolded.

Supplemental Table 7

Predicting Alcohol Use from Alcohol Use Polygenic Scores (MVP alc-PRS) and Social Support: Testing Developmental Differences Among European Americans Adults

|  | Emerging Adulthood  (n = 1056) | | | | Young Adulthood  (n = 1733) | | | | Middle Adulthood  (n = 1222) | | | | Wald Tests | |
| --- | --- | --- | --- | --- | --- | --- | --- | --- | --- | --- | --- | --- | --- | --- |
|  | B | SE | β | p | B | SE | β | p | B | SE | β | p | χ^2^  (df = 2) | p |
| **Step 1** |  |  |  |  |  |  |  |  |  |  |  |  |  |  |
| Sex | .70 | .06 | .32 | < .001 | .43 | .06 | .17 | < .001 | .52 | .07 | .22 | < .001 | -- | -- |
| Educational Attainment | -.04 | .02 | -.08 | .014 | -.08 | .02 | -.14 | < .001 | .00 | .02 | .01 | .843 | -- | -- |
| Household Income | .00 | .01 | .00 | .803 | -.04 | .02 | -.07 | .011 | -.02 | .01 | -.04 | .207 | -- | -- |
| Alc-PRS | .14 | .03 | .12 | <.001 | .08 | .03 | .06 | .015 | .08 | .04 | .07 | .017 | 2.03 | .363 |
| **Step 2** |  |  |  |  |  |  |  |  |  |  |  |  |  |  |
| Family Support | **-.15** | **.05** | **-.09** | **.004** | **.13** | **.05** | **.07** | **.017** | **.02** | **.07** | **.01** | **.805** | **14.30** | **<.001** |
| Friend Support | **.17** | **.07** | **.09** | **.014** | **-.22** | **.06** | **-.10** | **<.001** | **-.19** | **.07** | **-.09** | **.008** | **19.00** | **< .001** |
| **Step 3** |  |  |  |  |  |  |  |  |  |  |  |  |  |  |
| Alc-PRS x Family Support | -.14 | .05 | -.09 | .005 | .05 | .05 | .03 | .360 | -.01 | .06 | -.01 | .887 | 7.26 | .027 |
| Alc-PRS x Friend Support | .06 | .06 | .03 | .341 | -.06 | .06 | -.03 | .283 | -.01 | .07 | -.01 | .853 | 1.98 | .371 |

Note. Alc-PRS = alcohol consumption genome-wide polygenic score calculated using GWAS estimates from MVP EA sample. Emerging adulthood ages 18-29, young adulthood ages 30-44, and middle adulthood ages 45-65. Coefficients that are statistically significant at p < .025 across age groups are bolded.

Supplemental Table 8

Predicting Alcohol Use from Alcohol Use Genome-wide Polygenic Scores (MVP alc-PRS) and Social Support: Testing Sex Differences among European American Adults

|  | Males (N = 1824) | | | | Females (N = 2187) | | | | Wald Test | |
| --- | --- | --- | --- | --- | --- | --- | --- | --- | --- | --- |
|  | B | SE | β | p | B | SE | β | p | χ^2^ (df = 1) | p |
| **Step 1** |  |  |  |  |  |  |  |  |  |  |
| Age | -.02 | .00 | -.21 | < .001 | -.02 | .00 | -.21 | < .001 | -- | -- |
| Educational Attainment | -.05 | .02 | -.08 | .023 | -.03 | .01 | -.05 | .028 | -- | -- |
| Household Income | -.03 | .01 | -.06 | .027 | -.00 | .01 | -.01 | .609 | -- | -- |
| Alc-PRS | .12 | .03 | .09 | < .001 | .13 | .02 | .13 | < .001 | .05 | .822 |
| **Step 2** |  |  |  |  |  |  |  |  |  |  |
| Family Support | .09 | .06 | .04 | .133 | -.00 | .04 | .00 | .989 | 1.71 | .191 |
| Friend Support | -.20 | .06 | -.08 | .002 | -.06 | .05 | -.04 | .181 | 3.03 | .082 |
| **Step 3** |  |  |  |  |  |  |  |  |  |  |
| Alc-PRS x Family Support | -.03 | .06 | -.01 | .614 | -.02 | .05 | -.01 | .626 | .02 | .880 |
| Alc-PRS x Friend Support | .03 | .07 | .01 | .658 | -.04 | .07 | -.02 | .376 | .80 | .371 |

Note. Alc-PRS = alcohol consumption genome-wide polygenic score calculated using GWAS estimates from MVP EA sample.

Supplemental Table 9

Predicting Alcohol Use from Alcohol Use Polygenic Scores and Social Support: Testing Developmental Differences Among European Americans Adults with Smaller Age Bands

|  | Age 18-21 | | Age 22-29 | | Age 30-37 | | Age 38-44 | | Age 45-55 | | Age 56-65 | |  |  |
| --- | --- | --- | --- | --- | --- | --- | --- | --- | --- | --- | --- | --- | --- | --- |
|  | *β* | *p* | *β* | *p* | *β* | *p* | *β* | *p* | *β* | *p* | *β* | *p* | χ^2^  (df = 2) | p |
| **Step 1** |  |  |  |  |  |  |  |  |  |  |  |  |  |  |
| Sex | .33 | <.001 | .31 | <.001 | .20 | <.001 | .15 | <.001 | .26 | <.001 | .12 | .015 | -- | -- |
| Educational Attainment | .05 | .385 | -.07 | .116 | -.15 | <.001 | -.12 | .001 | -.01 | .746 | .10 | .086 | -- | -- |
| Household Income | .05 | .305 | -.04 | .280 | -.06 | .177 | -.07 | .057 | -.05 | .295 | -.02 | .598 | -- | -- |
| Alc-PRS | .13 | .013 | .09 | .026 | .11 | <.001 | .09 | .009 | .08 | .027 | .18 | <.001 | 2.97 | .704 |
| **Step 2** |  |  |  |  |  |  |  |  |  |  |  |  |  |  |
| Family Support | **-.02** | **.829** | **-.20** | **.002** | **.20** | **.003** | **.071** | **.363** | **.04** | **.563** | **.06** | **.621** | **20.52** | **<.001** |
| Friend Support | **.23** | **.062** | **.11** | **.219** | **-.29** | **.001** | **-.17** | **.049** | **-.14** | **.083** | **-.32** | **.008** | **20.52** | **.001** |
| **Step 3** |  |  |  |  |  |  |  |  |  |  |  |  |  |  |
| Alc-PRS x Family Support | -.07 | .185 | -.01 | .744 | -.01 | .815 | .03 | .573 | -.03 | .404 | -.13 | .028 | 5.15 | .398 |
| **Alc-PRS x Friend Support** | **.08^a^** | **.230** | **-.08^a^** | **.088** | **-.13^b^** | **.001** | **-.06^a^** | **.197** | **.03^a^** | **.434** | **-.03** | **.591^a^** | **12.94** | **.024** |

Note. Alc-PRS = alcohol consumption genome-wide polygenic score. Coefficients that are statistically significantly different across age groups are bolded. Results from pairwise comparisons for GxE effects are presented by superscripts, the same superscript indicates no difference, and different superscripts indicate significant difference in the coefficients.

Supplemental Table 10

Predicting Alcohol Use from Alcohol Use Polygenic Scores and Social Support: Testing Developmental Differences Among African Americans Adults with Smaller Age Bands

|  | Age 18-21 | | Age 22-29 | | Age 30-37 | | Age 38-44 | | Age 45-55 | | Age 56-65 | |  |  |
| --- | --- | --- | --- | --- | --- | --- | --- | --- | --- | --- | --- | --- | --- | --- |
|  | *β* | *p* | *β* | *p* | *β* | *p* | *β* | *p* | *β* | *p* | *β* | *p* | χ^2^  (df = 2) | p |
| **Step 1** |  |  |  |  |  |  |  |  |  |  |  |  |  |  |
| Sex | .33 | <.001 | .23 | <.001 | .27 | <.001 | .20 | .001 | .27 | <.001 | .42 | <.001 |  |  |
| Educational Attainment | -.22 | .007 | -.07 | .275 | -.02 | .727 | -.15 | .021 | -.06 | .416 | .01 | .882 |  |  |
| Household Income | .11 | .073 | -.00 | .993 | -.11 | .109 | -.17 | .002 | -.23 | .003 | -.18 | .054 |  |  |
| Alc-PRS | -.09 | .184 | .04 | .474 | .05 | .376 | -.01 | .921 | .02 | .767 | .01 | .990 | 3.522 | .620 |
| **Step 2** |  |  |  |  |  |  |  |  |  |  |  |  |  |  |
| Family Support | -.04 | .602 | -.00 | .986 | -.08 | .199 | -.06 | .318 | .13 | .143 | -.10 | .430 | 4.84 | .435 |
| Friend Support | -.17 | .060 | -.11 | .219 | -.01 | .850 | -.03 | .616 | .25 | <.001 | -.09 | .532 | 7.90 | .162 |
| **Step 3** |  |  |  |  |  |  |  |  |  |  |  |  |  |  |
| Alc-PRS x Family Support | .04 | .610 | .03 | .740 | .12 | .081 | -.05 | .454 | .11 | .129 | .05 | .578 | 4.78 | .444 |
| Alc-PRS x Friend Support | **.11^a^** | **.177** | **-.13^b^** | **.055** | **.07^ab^** | **.253** | **.01^ab^** | **.928** | **-.16^b^** | **.004** | **-.28^b^** | **.007** | **19.23** | **.002** |

Note. Alc-PRS = alcohol consumption genome-wide polygenic score. Coefficients that are statistically significantly different across age groups are bolded. Results from pairwise comparisons for GxE effects are presented by superscripts, the same superscript indicates no difference, and different superscripts indicate significant difference in the coefficients.
